# Exposure to TiO_2_ Nanoparticles Increases *Listeria monocytogenes* Infection of Intestinal Epithelial Cells

**DOI:** 10.3390/nano10112196

**Published:** 2020-11-04

**Authors:** Maria Grazia Ammendolia, Barbara De Berardis, Linda Maurizi, Catia Longhi

**Affiliations:** 1 National Center of Innovative Technologies in Public Health, Italian National Institute of Health, Viale Regina Elena 299, 00161 Rome, Italy; barbara.deberardis@iss.it; 2 Department of Public Health and Infectious Diseases, “Sapienza” University of Rome, Piazzale Aldo Moro 5, 00185 Rome, Italy; lindamaurizi92@gmail.com (L.M.); catia.longhi@uniroma1.it (C.L.)

**Keywords:** titanium dioxide, nanoparticles, *Listeria monocytogenes*, intestinal cells, bacterial invasion and survival

## Abstract

Titanium dioxide nanoparticles (TiO_2_ NPs) are widely used in a variety of consumer products. Cellular exposure to TiO_2_ NPs results in complex effects on cell physiology that could impact biological systems. We investigated the behavior of *Listeria monocytogenes* in intestinal epithelial cells pre-treated with either a low or high (1 and 20 µg/cm^2^) dose of TiO_2_ NPs. Our results indicate that the pre-treated cells with a low dose became more permissive to listeria infection; indeed, both adhesion and invasion were significantly increased compared to control. Increased invasion seems to be correlated to cytoskeletal alterations induced by nanoparticles, and higher bacterial survival might be due to the high levels of listeriolysin O that protects *L. monocytogenes* from reactive oxygen species (ROS). The potential risk of increased susceptibility to *L. monocytogene*s infection related to long-term intake of nanosized TiO_2_ at low doses should be considered.

## 1. Introduction

Titanium dioxide nanoparticles (TiO_2_ NPs) are among the most frequently used nanoparticles in many areas of life, such as environmental protection, building engineering, medicine, agriculture, food and the cosmetic industry [1]. TiO_2_ is an n-type semiconductor material characterized by a refractive index in both the ultraviolet and visible band. It is a widely used material in photocatalytic applications of different products, such as paints, tanning creams, piezoelectric devices, dye-sensitized solar cells, and gas sensor devices [2]. Due to their photocatalytic properties, TiO_2_ NPs can rather easily generate reactive oxygen species (ROS), resulting in good antibacterial properties [3]. Titanium NPs also possess promising properties as agents for photodynamic therapy, drug carrier systems and radiosensitizers. Studies in these fields are mainly focused on doping and functionalization methods to improve the activity of TiO_2_ NPs and minimize side effects [4].

In food, TiO_2_ NPs are common supplements that enhance the white color and brightness of food products, including cheeses, sauces, skimmed milk, ice cream, and even confectionery and gum products as a coating [5,6,7,8,9]. Its content in candy, chewing gum, chocolate and white-coated products is very high, reaching 2.5 mg Ti/g [5,8].

TiO_2_ NP applications in the food industry give rise to many controversies surrounding food safety. According to the European Food Safety Authority (EFSA), who, in 2016, performed the latest study on the safety of E171 (titanium dioxide), concentration values and exposure of humans to TiO_2_ NPs through food do not raise concerns. However, the International Agency for Research on Cancer (IARC) classified TiO_2_ as a potential carcinogenic agent, based on animal models of inhalation toxicity tests [10,11,12]. In addition, some studies have shown that nanoparticles can cause macro- and microelement deficiencies leading to digestive disorders an d inadequate absorption of food components [13]. After oral exposure, NPs enter the gastrointestinal tract, a significant absorption route for TiO_2_ NPs. In vivo tests have revealed that TiO_2_ NPs, taken orally or by inhalation, accumulate in the lungs, digestive tract, liver, heart, spleen, kidneys and cardiac muscle [14]. It has been suggested that TiO_2_ NPs might translocate through both the epithelium of ileum and Peyer’s patches, leading to damage and most likely chronic failure of the intestinal epithelium [15], as also observed in the small intestine of mice after oral gavage administration of TiO_2_ NPs [16]. Previously, we demonstrated [17] that TiO_2_ NPs, orally administered at a low dose in rats, entered the intestinal mucosa and influenced the male endocrine system, driving a higher testosterone production probably responsible for increasing villi sizes. Additionally, gastrointestinal nanoparticle exposure evidenced hepatic histopathologic damage and myocardial injury [18], as well as toxic damage in the mouse brain [19,20]. The endocrine system could also be influenced by nanosized Ti, TiO_2_ modulation of the reproductive-endocrine-immune system after repeated oral administration in adult rats was demonstrated [21].

TiO_2_ NPs toxicity was also studied for antimicrobial activity due to a strong oxidative potential caused by UV activation. However, the food additive and drug delivery application [22] do not require activation. In the absence of UV irradiation, TiO_2_ is reported to be toxic primarily to eukaryotic cells and not to bacteria [23,24,25], except at very high concentrations [26]. A possible concern could be when the cells are exposed to particles and bacteria at the same time.

Among foodborne bacteria, *Listeria monocytogenes* represent a severe public health and food safety problem [27,28,29,30]. These organisms target individuals with weakened immune systems, such as pregnant women, newborns, elderly persons, and immunocompromised hosts. The adaptation of *L. monocytogenes* depends on the procession of several well-coordinated molecular events essential for counteracting host defenses and facilitating the infection of different host cells, including the intestinal epithelial cells and macrophages [31].

Previously, we reported that the interaction between non-UV irradiated TiO_2_ NPs and *L. monocytogenes* led to increased biofilm formation [32], whereas bacterial internalization in intestinal cells was reduced with increasing nanoparticle concentrations. Since both TiO_2_ NPs and *Listeria monocytogenes* are conveyed through food, we suggested that increased biofilm formation in the gut could protect bacteria from the action of antimicrobial agents; moreover, planktonic cells may be continuously shed from the biofilm to re-infect the same host or be transmitted.

At present, there are no data concerning the modulation of pathogenic bacterial infection by nanosized material adsorbed by the intestinal mucosa. The daily intake of TiO_2_ NPs was estimated to be high, since this nanomaterial is present in different foods; thus, it is reasonable to hypothesize that continuous intestinal exposure to TiO_2_ NPs could create a microenvironment favorable for bacterial infections.

To verify whether TiO_2_ NPs could affect bacterial infection efficiency, we investigated the invasiveness of *Listeria monocytogenes* in intestinal epithelial cells, previously treated with TiO_2_ NPs.

## 2. Materials and Methods

### 2.1. Nanoparticles Suspensions

TiO_2_ anatase NPs with primary size <25 nm (Sigma-Aldrich Company Ltd., Gillingham, Dorset, UK) were dispersed in either Milli-Q water or Dulbecco’s modified minimum essential medium (DMEM) (HyClone Laboratories, South Logan, UT, USA) without serum. The suspensions were sonicated with a probe sonicator (Vibracell, Sonics & Materials Inc, Newtown, CT, USA, 750 W, 20 kHz, 20% amplitude, 6.5 mm probe diameter) under temperature-controlled conditions for 13 min to reduce agglomeration.

### 2.2. Characterization

TiO_2_ NP suspensions (36 μg/mL) were characterized by dynamic light scattering (DLS) to determine size distribution, agglomeration state, and surface charge. Then, 1 mL suspensions were analyzed by a Zetasizer Ultra (Malvern Instruments, Malvern, UK), and 10 measurements were performed for each of them. Equilibration step at 37 °C was set at 2 min, read number and duration of each measurement were set on automatic. Intensity distribution data were considered. The hydrodynamic diameter (Z-average) and polydispersity index (PDI) were determined by ZS Xplorer Software (Malvern Instruments, Malvern, UK).

Zeta potential measurements of TiO_2_ NP suspensions in DMEM (pH 7.4) were performed to assess NP preparation stability and surface charge. The measurements were conducted in triplicate on 750 μL of NP suspension using an automatic measurement protocol of Zetasizer Ultra.

Moreover, TiO_2_ NPs were characterized by electron microscopy using transmission electron microscopy (TEM) (EM 208, FEI Company, Eindhoven, The Netherlands) and scanning electron microscopy (SEM) (FE-SEM Quanta Inspect, FEI Company, Eindhoven, Netherlands) equipped with a Soft Imaging System to determine the shape, primary size, size distribution and agglomeration status as described by Ammendolia et al. (2014) [32].

### 2.3. Bacterial Strain

*Listeria monocytogenes* EGD-e strain [33] (serogroup 1/2a) were obtained from the American Type Culture Collection (ATCC) (Manassas, VA, USA). Bacteria were cultured in brain heart infusion (BHI) agar (DIFCO Laboratories, Franklin Lakes, NJ, USA) and incubated at 37 °C for 24 h. The overnight culture of the strain was prepared by inoculation of 6 mL of BHI broth with a sterile loop of the working stock and incubated at 37 °C for 18–20 h to achieve a cell load of 1 × 10^8^ Colony Forming Units (CFU)/mL. Hemolytic activity was measured as described by Alonzo et al. (2009) [34]. Hemolytic units (HU) were calculated after setting 0 HU as a negative control activity, and 100 HU for total hemolysis, observed in samples of erythrocytes lysed with 0.01% Sodium Dodecyl Sulfate (SDS).

### 2.4. Cell Line

Human colorectal adenocarcinoma cell line HT-29 cells were obtained from ATCC (Manassas, VA, USA) (catalog number: ATCC^®^ HTB-38™) and grown in DMEM (HyClone Laboratories, South Logan, UT, USA) supplemented with heat-inactivated fetal calf serum (FCS) (10% *v/v*), and L-glutamine (2 mmol/L). For the lactate dehydrogenase (LDH) and 3-(4,5-dimethylthiazol-2-yl)-2, 5-diphenyltetrazolium bromide (MTT) assays, HT-29 cells were seeded in 96-well plates and treated with different concentrations of TiO_2_ NPs. Before all experiments, to ensure dispersion of the particles, TiO_2_ NPs were predispersed in culture medium using ultrasound (mean potency/peak 90/180 W, +4 °C) for 45 min.

### 2.5. TiO_2_ NP Cytotoxicity

#### 2.5.1. The Lactate Dehydrogenase (LDH) Leakage Assay

The release of LDH was determined using the Cytotoxicity Detection Kit (Plus) (LDH) from Roche Diagnostics (Mannheim, Germany). Cells (20 × 10^4^ cells/mL), seeded in 24-well plates, were treated with TiO_2_ NPs (0, 1, and 20 μg/cm^2^, corresponding to 0, 1.8, and 36 μg/mL) for 6, 24, and 48 h. After time intervals, culture medium was collected, substrate solution added, and plates incubated for 30 min at room temperature. LDH release was measured with a microplate reader (Perkin-Elmer, Boston, MA, USA) by using a 490 nm wavelength. Each experiment was done in triplicate. Cytotoxicity was expressed as a percentage relative to the basal LDH release by untreated control cells set as 0%.

#### 2.5.2. The 3-(4,5-Dimethylthiazol-2-yl)-2,5-Diphenyltetrazolium Bromide (MTT) Assay

HT-29 cells (1 × 10^5^ cells/mL) were grown in 96-well microplates for 24 h in a 5% CO_2_ atmosphere and treated with NPs (0, 1 and 20 μg/cm^2^). After incubation at 37 °C for 6, 24 and 48 h, a MTT (Sigma Chemical Co., Saint Louis, MO, USA) solution (5 mg/mL) was added to each well, and plates were incubated for an additional 3 h. Dimethyl sulfoxide (100 μL) was added to each well, after removing supernatants, to dissolve formazan crystals. Optical density was determined at 570 nm (A570) with a spectrophotometer/fluorimeter microplate reader (Perkin-Elmer, Boston, MA, USA). Cell viability was expressed as a percentage of control untreated samples, set as 100%.

### 2.6. Bacterial Adhesion and Invasion after Cell Pre-Treatment with TiO_2_ NPs

Cells monolayers, treated with TiO_2_ NPs (0, 1, and 20 μg/cm^2^) for different times (6 and 24 h), were infected with bacteria for the adhesion and invasion assays, after washing away the excess of nanoparticles.

For the adhesion assay, bacterial cultures containing logarithmically grown bacteria at a Multiplicity of Infection (MOI) of 100 bacteria/cell were incubated with cells for 1 h at 37 °C. After this incubation period, cells were extensively washed, lysed with ice-cold 0.1% Triton X-100 and plated on tryptone soy agar (TSA) (DIFCO Laboratories, Franklin Lakes, NJ, USA) to determine bacterial adherence.

For the invasion assay, after washing, 1 mL of fresh medium containing 50 μg/mL of gentamicin was added to each well and maintained for 1 h at 37 °C. Cells were then lysed as for the adhesion assay.

Adhesion efficiency was expressed as the percent of the inoculated CFU that were recovered. The adhesion percentage of bacteria to HT-29 cells in the presence of NPs was evaluated with respect to the percentage of the adhesion of *L. monocytogenes* in the absence of NPs, considered as 100%.

Invasion efficiency was expressed as the percent of the inoculated bacteria that were recovered. The invasion percentage of bacteria to HT-29 cells in the presence of NPs was evaluated with respect to the invasion percentage of *L. monocytogenes* in the absence of NPs, considered as 100%.

### 2.7. Bacterial Survival after Cell Pre-Treatment with TiO_2_ NPs

For the intracellular growth assays, incubation of infected cells in the gentamicin-containing medium was prolonged for an additional period of 3 h and a further 24 h at 37 °C (with freshly added 15 μg/mL gentamicin), followed by lysis and CFU counts. Bacterial survival was expressed as the CFU percentage counted at 24 h post-infection divided by the CFU number after 3 h post-infection.

### 2.8. Measurement of Intracellular Reactive Oxygen Species (ROS) Production

Carboxy-2’,7’-dichlorodihydrofluorescein diacetate (H_2_DCFDA) (Molecular Probe, Eugene, OR, USA) was used to monitor the generation of ROS. HT-29 cells, incubated with TiO_2_ NPs (0, 1, and 20 μg/cm^2^) for 6 and 24 h under standard conditions, were treated with 40 μM carboxy-H_2_DCFDA for 30 min at 37 °C. Then, 2′,7′-dichlorofluorescein (DCF) fluorescence was measured at excitation/emission at 488/535 nm with a fluorescent microplate reader (Perkin- Elmer, Boston, MA, USA). Cells without nanoparticles and 100 μM H_2_O_2_ were used as negative and positive controls, respectively. Pre-treated cells with nanoparticles incubated for different times were also infected with EGD-e strain at an MOI of 100 bacteria/cell and evaluated for ROS production at 3 and 24 h post-infection, as described above.

### 2.9. Actin and E-Cadherin Staining

To visualize actin filaments, cells, grown in 8-chamber culture slides (Lab-Tek, Nunc, Thermo Fisher Scientific, Waltham, MA, USA) for 48 h at 37 °C in 5% CO_2_ atmosphere, were incubated with selected TiO_2_ NP concentrations (1, and 20 μg/cm^2^) for 2 and 6 h at 37 °C. Then, cells were fixed by incubating with 3.7% paraformaldehyde in Phosphate Buffered Saline (PBS) for 5 min at room temperature, washed with PBS and permeabilized by incubation with 100 μL of PBS containing 0.1% Triton X-100 for 10 min at room temperature. After washing with PBS, cells were incubated in the dark for 20 min at room temperature with fluorescein-5-isothiocyanate (FITC)-phalloidin (Sigma Chemical Co., Saint Louis, MO, USA) diluted into PBS. After extensive PBS washing, glass slides were mounted in glycerol and observed using an Olimpus BX53 microscope (Waltham, MA, USA), equipped with a Tucsen Camera GT H series and Tucsen ISCapture 5.1.1 software.

Additionally, HT-29 cells, treated as described above, were stained for E-cadherin expression using a rabbit polyclonal antibody against human E-cadherin (Santa Cruz Biotechnology, Inc., Dallas, TX, USA) and incubated at 37 °C for 40 min. After 1 h of further incubation with FITC-conjugated goat anti-rabbit IgG (Sigma Chemical Co., Saint Louis, MO, USA) cells were washed with PBS and mounted for fluorescence visualization.

### 2.10. Transmission Electron Microscopy (TEM)

Cells were plated in 12-well tissue culture plates and treated with 1 and 20 μg/cm^2^ of TiO_2_ NPs. After 2, 6 and 24 h of incubation, treated cells were fixed using 2.5% glutaraldehyde in sodium cacodylate buffer (0.1 M, pH 7.2) at room temperature for 1 h, post-fixed with 1% osmium tetroxide solution, dehydrated in graded ethanol, and embedded in epoxy resin. Ultra-thin 80 nm sections were cut, mounted on copper grids, and stained with lead citrate and saturated aqueous uranyl acetate. Samples were examined with a Philips 280S transmission electron microscope at 80 kV.

TiO_2_ NP exposed cells (2, 6, and 24 h) were also infected with logarithmically grown EGD-e (MOI: 100) and incubated for 1 h at 37 °C. Then, a gentamicin-containing medium was added for an additional incubation time of 3 h. Infected cells were then fixed and processed for electron microscopy, as described above.

### 2.11. Statistical Analysis

All results were calculated from data from at least three independent experiments and expressed as means ± standard deviation (SD). In all experiments, NP treated, and NP pre-treated infected samples were compared with respective controls. Statistical analyses were performed by one-way ANOVA followed by post hoc pairwise t-tests (Graph Pad Prism, Version 5.0). A *p* value of less than 0.05 (* *p* < 0.05) was considered significant.

## 3. Results

### 3.1. TiO_2_ NP Characterization

#### 3.1.1. Hydrodynamic Diameter

Characterization by DLS was performed on TiO_2_ NP suspensions at 36 μg/mL, corresponding to the high dose used in this study. Z-average and PDI and the results of the size distribution of NPs in Milli-Q water and DMEM without serum are displayed in Table 1. The size distribution of TiO_2_ NP suspensions in Milli-Q water showed the presence of two peaks: a peak with a higher intensity of the light scattered around 473 nm and a slower peak at 110 nm. The size distribution of NPs in the culture medium showed the presence of one peak around 1120 nm.

PDI values indicated that TiO_2_ NP suspensions both in Milli-Q water and in culture medium were polydisperse. The high Z-average value and the size distributions highlight large agglomerates in both NP suspensions. Moreover, NPs dispersed in DMEM increased their hydrodynamic diameter significantly, suggesting that the culture medium did not improve the polydispersity of suspensions.

#### 3.1.2. Surface Charge

The zeta potentials of TiO_2_ NPs were negative both in Milli-Q water and culture medium, as shown in Table 2. Moreover, the zeta potential for NPs in DMEM was lower in absolute value, indicating a higher instability of this suspension.

#### 3.1.3. Electron Microscopy Characterization

**Figure 1:**
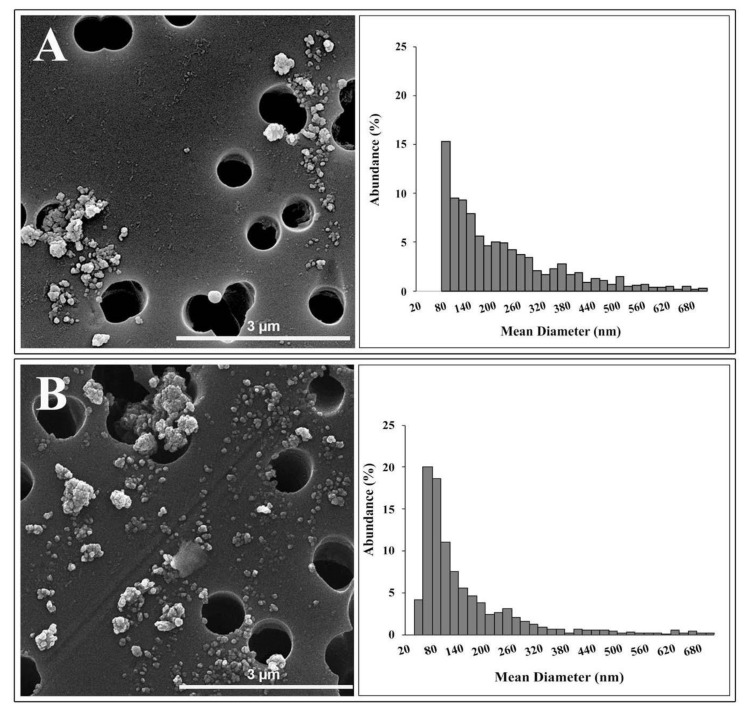
SEM agglomerates and mean diameter distribution of TiO_2_ NPs in Milli-Q water (**A**) and DMEM (**B**) determined by SEM analysis. The range of mean diameter between 0 and 700 nm is displayed.

TEM analysis identified two different morphologies in TiO_2_ NP samples: spherical and irregular shape. The primary size of spherical NPs ranged from 20 to 60 nm. Irregular shape showed a length of about 60 nm and a width of about 40 nm. SEM observations (Figure 1) displayed that NPs joined large agglomerates, up to 2 μm in length.

Figure 1 showed the granulometric spectrum of the TiO_2_ NPs in Milli-Q water (A) and in DMEM (B) at 36 μg/mL, respectively, determined by SEM analysis.

The size distribution of NPs in Milli-Q water ranged from 70 nm to 1.1 μm, with a peak between 60 and 80 nm. The average diameter of these NPs was about 70 nm, and their abundance was equal to 15.3%. Only 24.8% of NPs analyzed were below 100 nm.

The granulometric spectrum of TiO_2_ NPs in the culture medium showed a long tail ranging between 31 nm and 1.9 μm. The size distribution showed a peak in the range of 40–60 nm, with an abundance—20%—of particles. The NPs belonging to this granulometric range had an average diameter of about 48 nm. Finally, 53.8 % of NPs in the culture medium, analyzed by SEM, possessed dimensions below 100 nm. Results on size distribution agree with data available in the literature on the size distribution of the E171 food additive [9,35].

DLS and SEM analyses are in broad agreement. The characterization techniques show that the NPs suspended in Milli-Q water are strongly agglomerated, as indicated by both the values of hydrodynamic diameter and PDI of the suspensions obtained by DLS and by the absence of particles smaller than 70 nm and the long tail of the size distribution obtained by SEM. Furthermore, the polydispersion and agglomeration of TiO_2_ NPs do not seem to improve significantly when the NPs are suspended in DMEM, as suggested by the increased hydrodynamic diameter, the lower zeta potential and longer tail of the size distribution up to 1.9 μm, obtained by SEM. SEM size distribution of NPs in DMEM showed an abundance of particles or agglomerates—higher than 50%—with a mean diameter of less than 100 nm. This disagreement with DLS data could be due to different states in which NPs are found in the two analytical techniques, and the different physical phenomena used to determine size distribution and artifacts during SEM sample preparation. Moreover, since both DLS and SEM analyses showed the presence of NP agglomerates greater than 1 µm, the intensity of the light scattered by particles less than 100 nm could be covered by the intensity resulting from the large agglomerates because of the scattering intensity dependence on the sixth power of the particle radius [36].

### 3.2. TiO_2_ NP Cytotoxicity

**Figure 2:**
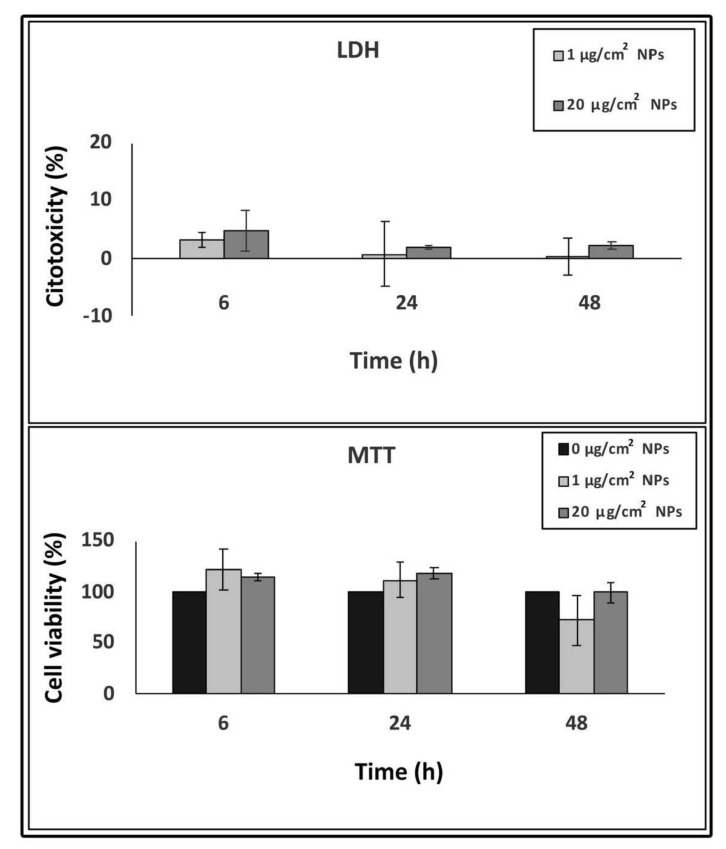
Concentration and time-dependent cytotoxicity of TiO_2_ NPs in HT-29 cells measured by lactate dehydrogenase (LDH) and 3-(4,5-dimethylthiazol-2-yl)-2, 5-diphenyltetrazolium bromide (MTT) assays. Data are presented as % of the control (taken as 0% cytotoxicity for LDH and 100% cell viability for MTT) with means ± SD calculated from three independent experiments.

To evaluate cell damage after TiO_2_ NP treatment, we performed LDH and MTT assays. LDH is an enzyme released into the culture medium upon damage of the plasma membrane and provides information on the cell membrane integrity. In our experiments, LDH content in the culture medium of TiO_2_ NP treated samples did not reveal significant cytotoxic effects compared to the control untreated samples, after 6, 24 and 48 h treatment (Figure 2).

The MTT assay was used to evaluate cell viability because of the capacity of the enzymes from the viable cells to reduce the MTT salt to formazan. As for the LDH assay, cell viability results, directly proportional to the amount of obtained formazan, show no statistically significant values between TiO_2_ NP treated cells and control samples, at all interval times, indicating no viability reduction by TiO_2_ NPs (Figure 2).

No interactions were detected between dyes and NPs in a cell free system (data not shown).

### 3.3. Bacterial Adhesion and Invasion in TiO_2_ NP Pre-Treated Cells

**Figure 3:**
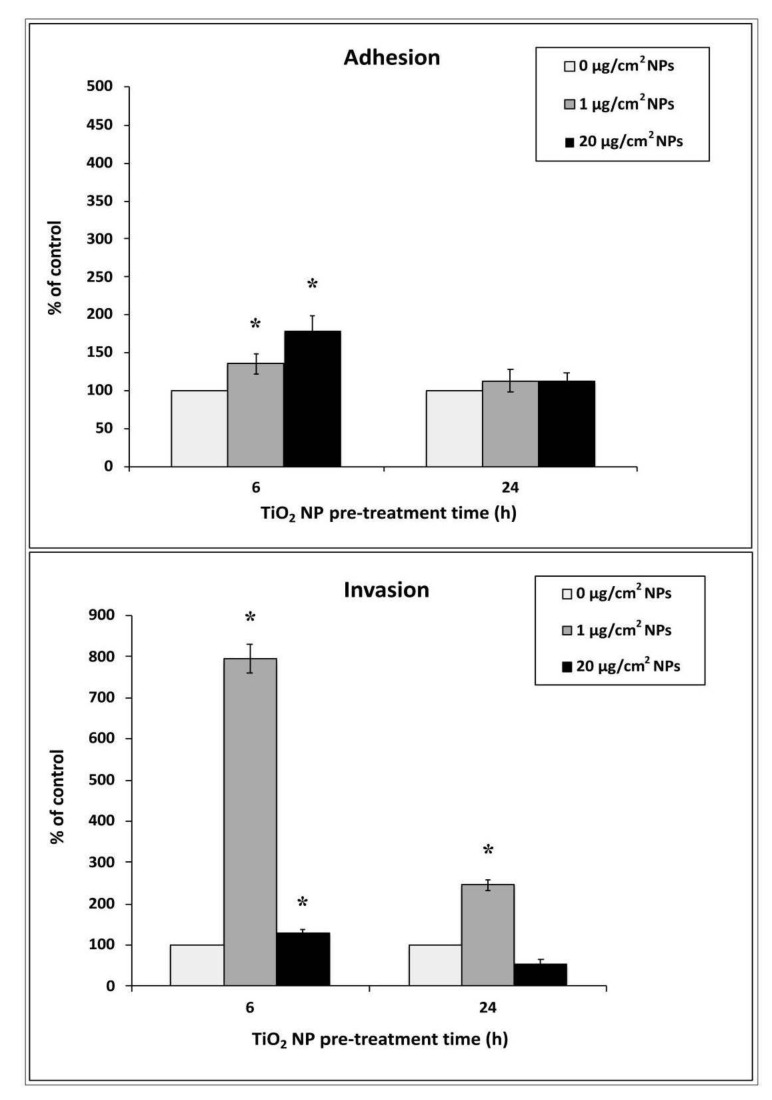
Adhesion and invasion efficiency of EGD-e strain in HT-29 cells pre-treated with TiO_2_ NPs at either low or high doses. Values are expressed as a percentage of control untreated infected cells (taken as 100%) with means ± SD calculated from three independent experiments. Asterisks denote statistically significant values compared to control untreated infected cells (* *p* < 0.05).

The capability of EGD-e cells to both adhere to and enter HT-29 cells pre-treated with 1 and 20 μg/cm^2^ of TiO_2_ NPs was assayed. Based on CFU counts (Figure 3), HT-29 cells pre-treated with both doses of TiO_2_ NPs showed, after 6 h treatment, a significantly higher percentage of adherent bacterial cells (in a dose-dependent manner) than control cells that were exposed to *L. monocytogenes* but were not pre-treated with NPs (135.5 ± 13.4 for 1 μg/cm^2^ and 179.3 ± 20 for 20 μg/cm^2^, respectively, * *p* < 0.05). At 24 h of treatment, adhesion values appeared similar, for both doses, to control infected cells without NP pre-treatment (113.15 ± 15 for 1 μg/cm^2^ and 112.8 ± 10.3 for 20 μg/cm^2^).

Regarding the invasion assay, cells pre-treated with both doses for 6 h of treatment showed an increased bacterial internalization with values dramatically high for 1 μg/cm^2^, with CFU raising to about eight times (795 ± 35, * *p* < 0.05) compared to control infected and NP untreated cells. When HT-29 cells were treated for 24 h with a low dose, bacterial invasion resulted in being about 2.5 times higher with respect to control cells, whereas cells pre-treated with NP high dose showed a trend to decreased invasion (245.5 ± 13 for 1 μg/cm^2^ and 53 ± 12 for 20 μg/cm^2^, respectively, * *p* < 0.05).

### 3.4. Bacterial Survival in TiO_2_ NPs Pre-treated Cells

**Figure 4:**
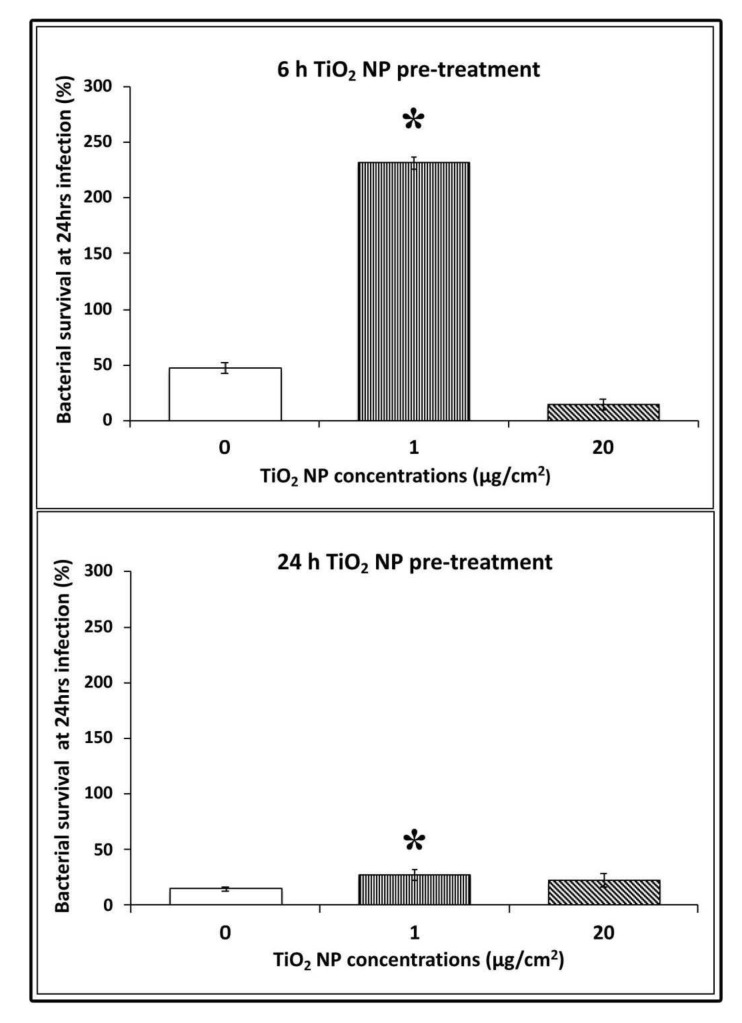
Bacterial survival after 24 h of infection in HT-29 cells pre-treated with TiO_2_ NPs. Values corresponded to the number of CFU 24 h post-infection divided by the number of CFU 3 h post-infection. Data are presented as means (± standard deviation) of at least three experiments. Asterisks indicate statistically significant values compared to control untreated infected cells (* *p* < 0.05).

To test the survival capability of *L. monocytogenes* in NP pre-treated cells, we compared bacterial counts at 24 h post-infection against 3 h of infection, which was considered the interval time of the completed bacterial invasion. At 6 h of NP pre-treatment, bacterial survival was significant only for low doses with CFU counts, which were 4.5 times higher than control infected cells (CFU counts 231.4 ± 20.7 vs. 14.5 ± 5, respectively; * *p* < 0.05). This indicates that *L. monocytogenes* were able to survive and multiply in these conditions more efficiently. Whereas at high doses of pre-treatment, the survived cells decreased considerably (more than half with respect to control uninfected cells; 14.39 ± 8.7 vs. 47.2 ± 5; * *p*< 0.05). At 24 h pre-treatment, bacterial survival was also higher for low dose with values considerably smaller with respect to 6 h pre-treatment (22 ± 4.2 vs. 14.59 ± 2.4, respectively; * *p* < 0.05) (Figure 4).

### 3.5. Intracellular ROS Generation

To measure ROS production induced by TiO_2_ NP treatment, we performed the DCF fluorescent assay. 2′,7′-dichlorodihydrofluorescein diacetate (H_2_DCFDA) diffuses into cells and, after deacetylation by cellular esterases, forms 2′,7′-dichlorodihydrofluorescein (H_2_DCF). In the presence of ROS, H_2_DCF is rapidly oxidized to the highly fluorescent compound 2′,7′-dichlorofluorescein (DCF), which provides a ROS production measure.

**Figure 5:**
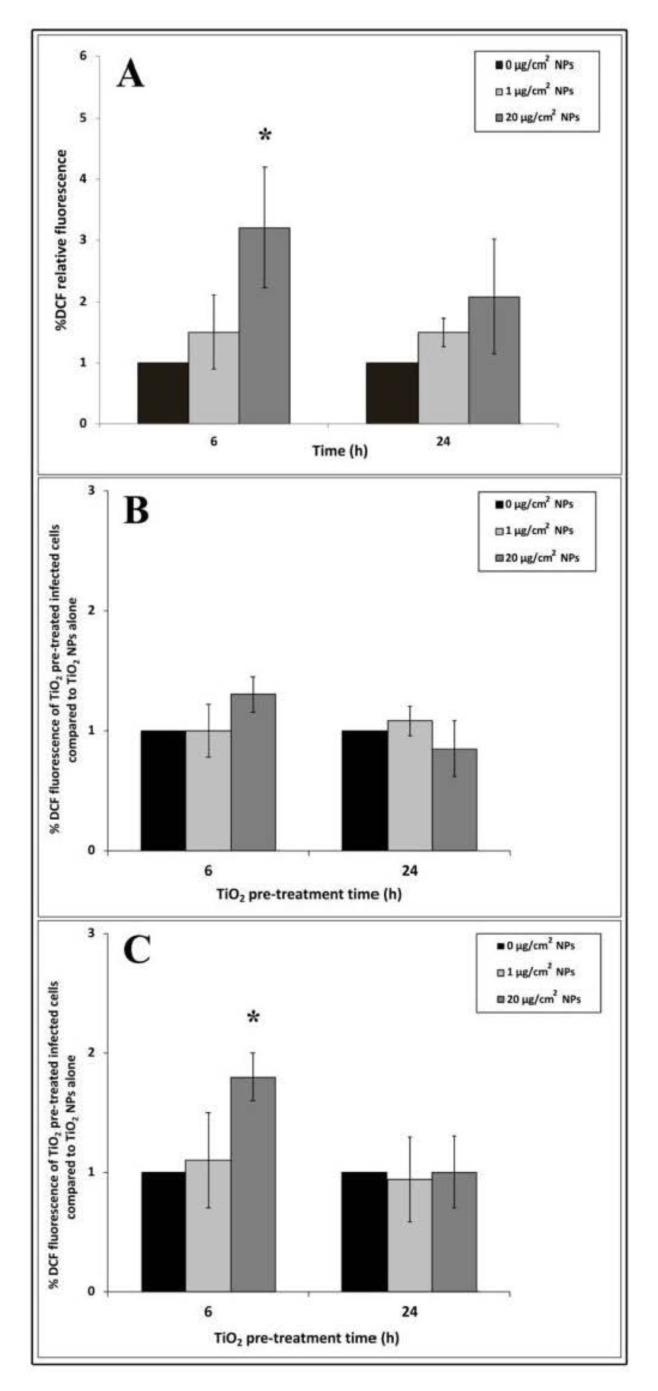
Reactive oxygen species (ROS) measurements in HT-29 cells pre-treated with TiO_2_ NPs alone and following EGD-e infection. (**A**): TiO_2_ NPs alone. (**B**): 3 h of EGD-e infection. (**C**): 24 h of EGD-e infection. Values are expressed as a percentage of the control samples (taken as 1%) with means ± SD calculated from three independent experiments. Asterisks denote statistically significant values compared to control untreated cells for Panel A and control NP untreated infected cells for Panels B and C (* *p* < 0.05).

An increasing trend of intracellular oxidative stress after 6 h of NP exposure was revealed by DCF fluorescence intensity (Figure 5A). The ROS level increased with the increase in nanoparticle concentrations but only 20 μg/cm^2^ induced significant differences compared to untreated cells. Compared to 6 h, DCF fluorescence intensity appeared to decrease at 24 h interval time in all titanium exposure samples.

To explore changes in ROS production due to EGD-e cell infection, we compared NP treated and infected samples with samples treated with NP alone by the ratio of their DCF fluorescence values. Cells treated with 1 μg/cm^2^ for 6 h and infected with listeria cells showed no significant increase in oxidative stress values after 3 and 24 h of infection compared to control infected cells (Figure 5B,C). Reactive oxygen species showed a trend towards increasing with high dose after 6 h of TiO_2_ NP pre-treatment followed by 3 h of listeria infection, even though no significant values compared to control were reported (Figure 5B). Significant values (* *p* < 0.05) were observed in the same TiO_2_ NP pre-treated time samples infected for 24 h. Regarding samples pre-treated with TiO_2_ for 24 h and infected for 3 and 24 h, ROS production was comparable to control cells challenged with EGD-e alone (Figure 5B,C).

### 3.6. Actin and E-Cadherin Fluorescence

To assess the possible activity of TiO_2_ NPs on cytoskeleton components responsible for an increased bacterial entry, we performed staining of actin using FITC-phalloidin, which is commonly used to selectively label actin because of their strong affinity for filamentous actin (F-actin).

**Figure 6:**
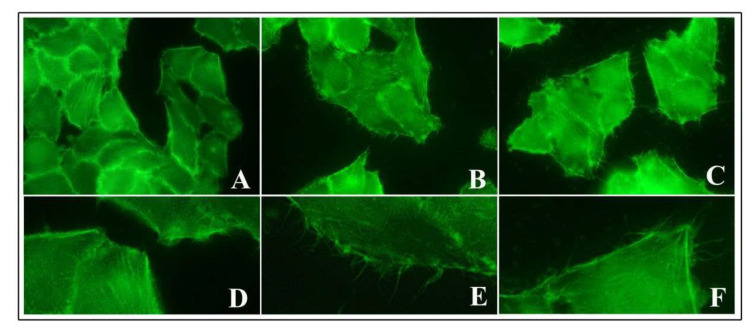
Actin phalloidin staining of HT-29 cells pre-treated with TiO_2_ NPs. Representative images of control untreated cells (**A**,**D**), pre-exposed cells to 1 μg/cm^2^ (**B**,**E**), and 20 μg/cm^2^ (**C**,**F**) at 6 h of NP treatment. Magnification: 400× for (**A**–**C**) and 4200× for (**D**–**F**).

We observed that the untreated control cells showed a basal amount of protrusive structures, which were positive for phalloidin staining from membrane surfaces (Figure 6A,D). At 2 h of NP exposure, these structures increased significantly after both low and high doses of NPs. Cells developed numerous long, thin and thick filopodia, protruding from the edge of cellular margins and extending towards adjacent cells or substrate (Figure 6B,E for 1 μg/cm^2^ and Figure 6C,F for 20 μg/cm^2^). Microscopic observations indicated that these filopodial structures were also more prolonged than those of control cells. A similar picture was observed at 6 h of NP treatment.

We performed E-cadherin labelling to explore *Listeria* receptor expression in intestinal cells, but no differences were visualized between control untreated and NP treated cells (data not shown).

### 3.7. Electron Microscopy

Transmission electron microscopy was performed to visualize ultrastructural changes in HT-29 cells treated with TiO_2_ NPs and infected with EGD-e.

**Figure 7:**
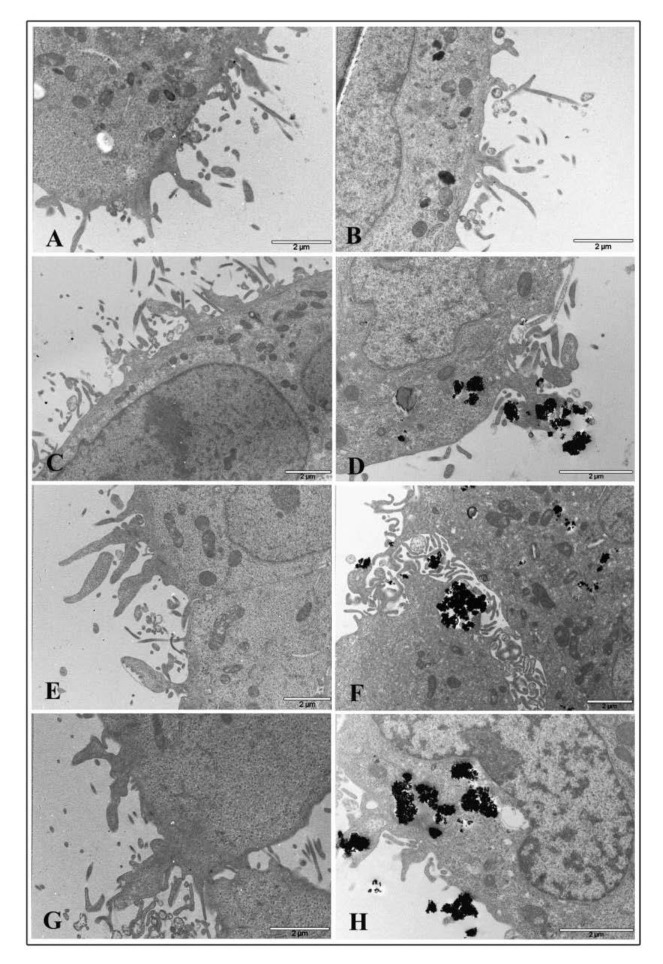
Transmission electron microscopy micrographs of HT-29 cells following exposure to TiO_2_ NPs. Representative images of control untreated cells (**A**,**B**) following 1 μg/cm^2^ treatment at 2 h (**C**), 6 h (**E**), and 24 h (**G**) time exposure, and after 20 μg/cm^2^ treatment at 2 h (**D**), 6 h (**F**), and 24 h (**H**) time exposure.

Micrograph images of TiO_2_ NP pre-treated intestinal cells showed no relevant damage to cytoplasmic organelles at all interval times both with 1 and 20 μg/cm^2^ concentrations. Ultrastructural alterations appeared on cell membranes with many surface protrusions at both NP concentrations. Compared to control (Figure 7A for 1 μg/cm^2^ and Panel B for 20 μg/cm^2^), showing some filamentous protrusions, NP treated cells revealed more membrane extensions similar to filopodia or lamellipodia. However, TiO_2_ NP treated cells were visualized to bind and enclose nanoparticle agglomerates at NP higher concentrations (Figure 7C,E,G for 1 μg/cm^2^ and Figure 7D,F,H for 20 μg/cm^2^, respectively).

**Figure 8:**
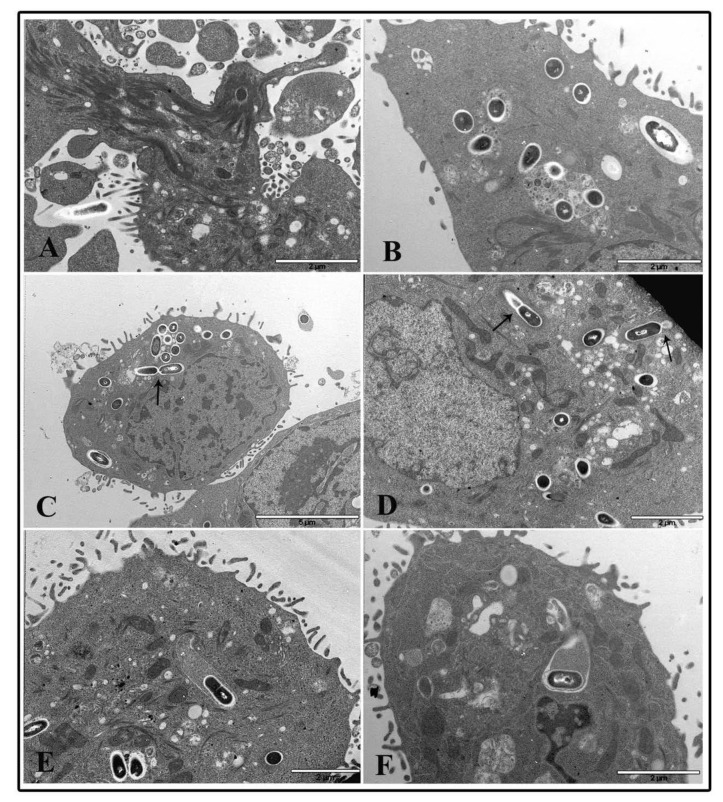
Transmission electron microscopy micrographs of HT-29 cells following exposure to 1 μg/cm^2^ TiO_2_ NPs and infected with *L. monocytogenes*. Representative images of TiO_2_ NP pre-treated cells at 2 h (**A**,**B**) and 6 h (**C**–**F**).

Cytoskeletal alterations were more pronounced in NP treated cells infected with *L. monocytogenes*. At 1 μg/cm^2^ after 2 h of treatment and listeria infection, dramatic actin movement was observed with marked changes in actin dynamics that led many bacterial cells to enter pre-treated cells (Figure 8A,B). Samples pre-treated for 6 h with NP low dose and infected with *L. monocytogenes* for 3 h showed a lot of bacterial cells both inside phagosomes and free in the cytoplasm surrounded by actin coat due to actin polymerization by listeria after vacuole escape (Figure 8C–F). Bacterial cells inside phagosomes showed structural integrity, and some of those free in the cytoplasm also displayed division activity (Panel C and D, arrows). Starting at 6 h of TiO_2_ NP treatment, almost all bacterial cells were visualized free in the cytoplasm and coated with actin, together with coated bacteria enclosed into vacuoles, indicating that most of the bacteria can pass cell-to-cell (Figure 8D–F). A similar picture was visualized at 24 h of TiO_2_ NP pre-treatment (data not shown).

**Figure 9:**
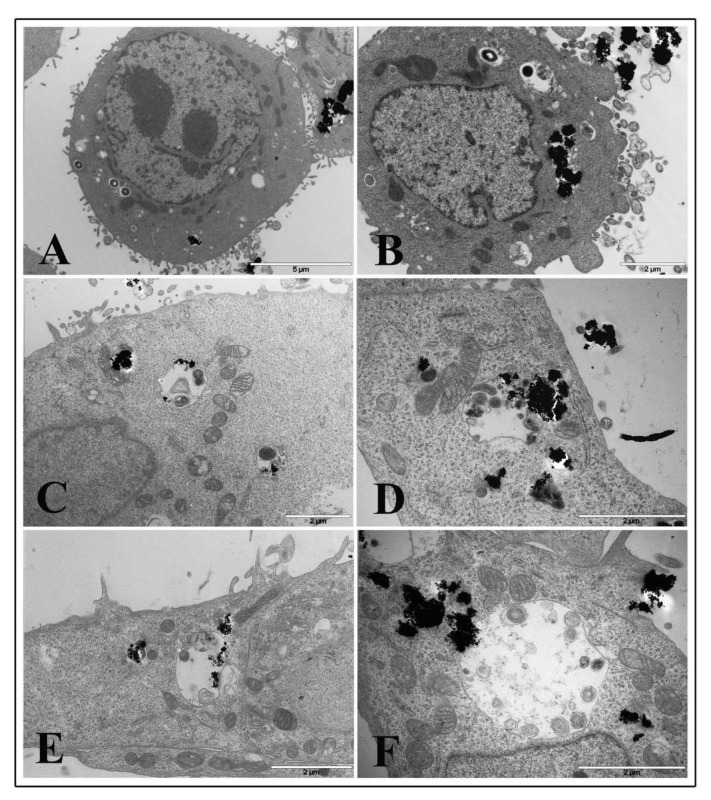
Transmission electron microscopy micrographs of HT-29 cells following exposure to 20 μg/cm^2^ TiO_2_ NPs and infected with *L. monocytogenes*. Representative images of TiO_2_ NP pre-treated cells at 2 h (**A**,**B**) and 6 h (**C**–**F**).

On the contrary, cells pre-treated with the high TiO_2_ NP concentration at the 2 h time interval showed few bacteria inside phagosomes and a large amount of nanoparticle agglomerates inside cytoplasmic vacuoles or free in the cytoplasm (Figure 9A,B). A few bacterial cells were found free in the cytoplasm. At 6 h of NP treatment, it was more challenging to visualize bacteria inside cells, whereas many vacuoles containing organelles and presumably bacterial bodies in an advanced and damaged state were revealed (Figure 9C–F).

Curiously, it seems that nanoparticle aggregates appeared to drive towards degrading vacuoles (Figure 9E,F). A similar picture was observed at 24 h of TiO_2_ NP treatment and infection (data not shown).

## 4. Discussion

Exposure to TiO_2_ NPs is likely to occur in conjunction with other pathogenic impacts, such as microbial infections. Since TiO_2_ NPs and *L. monocytogenes* were both vehiculated through foods, their interaction may trigger reactions at the gut level and produce additional damaging effects. In this study, sequential exposure of intestinal cells to TiO_2_ NPs and *L. monocytogenes* appeared to induce modifications leading to increased bacterial infection. Major cell permission to bacterial infection was observed when monolayers were pre-treated with a low dose of TiO_2_ NPs, a concentration comparable to that reported for human ingestion, which is approximately 0.03–3 mg/kg bw/day [5,37,38,39].

*L. monocytogenes* use a variety of bacterial proteins called internalins to invade nonphagocytic epithelial cells. Internalin A, a bacterial cell wall-anchored protein, binds to the epithelial host cell receptor E-cadherin, which mediates a physical link between the bacterium and filamentous actin (F-actin).

We have demonstrated that TiO_2_ NPs at low concentrations, such as 5 μg/cm^2^, were capable of inducing the expression of E and P selectins and ICAM-1, VCAM-1 and PECAM-1 in endothelial cells [40]; recently, Rueda-Romero et al. (2016) [41] have demonstrated that TiO_2_ NPs at low concentrations could activate human monocytes by heightening the expression of adhesion molecules ligands. In an attempt to explain the increased number of intracellular bacteria, we performed cell membrane staining for E-cadherin. We did not observe a significant difference between the TiO_2_ NP treated and the untreated cells regarding the amount of membrane E-cadherin expression.

Although the interactions between internalin A and E-cadherin are critical for *L. monocytogenes* epithelial cell invasion in vitro [42] and in vivo [43], it seems that the entire E-cadherin/catenin/F-actin complex is not always required for invasion. Ortega et al. (2017) [44] demonstrated that depleting αE-catenin or expressing truncated E-cadherin prevented *L. monocytogenes* from interacting with F-actin and had only mild effects on the efficiency of bacterial entry in epithelial cells. Therefore, in addition to this system, *L. monocytogenes* can use alternative modes of entry into epithelial cells.

Despite TiO_2_ NP pre-treatment did not increase listeria intestinal cell receptors, actin fluorescent staining of NP pre-treated monolayers showed cytoskeletal modifications, which were not observed in the control cells. At either low or high doses, cells showed an increased amount of membrane surface extensions, also visualized by electron microscopy. Specifically, we observed projections of plasma membranes, such as filopodial or lamellipodial protrusions, which, at a high dose, appeared to reach NPs and enclose them. From our observations, these projections do not seem to quantitatively differ between the low and high NP doses, despite the difference in bacterial invasion levels. When monolayers were pre-treated with a low dose of NPs and infected with *L. monocytogenes,* cytoskeletal changes became more pronounced with dramatic modifications in the dynamics of actin filaments, which led many bacteria to be internalized by cells.

Previous works have evidenced an alteration of calcium homeostasis in different cell types after exposure to TiO_2_ NPs [45,46]. Changes in intracellular calcium levels mediate various cellular processes, such as the reorganization of the cytoskeleton, focal adhesion turnover, invadopodia formation, actomyosin contractility, and triggering the development of lamellipodia [47]. Phagocytic processes are also driven by a finely controlled rearrangement of the actin cytoskeleton, where calcium plays an essential role by either controlling the activities of several contractile proteins or activating intracellular signaling pathways.

In our system, cytoskeletal changes could be due to calcium modulation by TiO_2_ NPs, which likely affects the cellular pathway in both titania doses. It has been demonstrated that a minimal intracellular content of titanium is necessary to induce a marked calcium homeostasis alteration [48]. Thus, it is reasonable to hypothesize that a low dose of NPs is sufficient to affect biological pathways regulating calcium influx or release from endoplasmic reticulum calcium stores, consequently impacting cytoskeletal proteins. This mechanism was not amplified in high dose pre-treated samples because of other factors, such as aggregation status, which decreases nanoparticle internalization and then the real intracellular NP concentration.

Our adhesion and invasion assays indicate that bacterial cell adhesion and entry efficiency were reduced at NP high dose compared to low dose. These results could be explained by membrane stiffness due to NP large agglomerates, which induce modification of membrane fluidity. Anatase titanium has a high number of hydroxyl groups (-OH) on its surface [49]. Hydroxyl groups lead to a higher binding of nanoparticles to the cell membranes, thereby increasing cellular membrane rigidity [50]. da Rosa (2013) [51] observed increased stiffness of the membrane in neutrophils treated with TiO_2_ NPs. It is well known that negatively charged TiO_2_ particles bind preferentially to amino acids with -OH, -NH, and -NH_2_ in their side chains [52]. Our data on TiO_2_ NP characterization in DMEM have shown that NPs joined together in large agglomerates and were negatively charged. Thus, TiO_2_ NPs can possibly impair cell membrane function, this was also observed by Xu et al. (2016) [53], who showed that HeLa cell membranes were significantly harder after exposure to 100 μg/mL titanium anatase nanoparticles with reducing *Staphylococcus aureus* binding and entry.

The large number of bacteria internalized in cells pre-treated with a low dose of TiO_2_ NPs exhibit a remarkable ability to avoid cell degradation mechanisms showing a high grade of survival also at 24 h post-infection compared to 3 h post-infection. It is known that *L. monocytogenes* are not only able to inhibit phagosome–lysosome fusion [54] but also actively avoid autophagic degradation [55,56]. Upon entry into host cells, bacteria can escape from the phagosome through the activity of three virulence factors: listeriolysin O (LLO), a phosphatidylinositol-specific phospholipase C (PI-PLC), and a broad-range phospholipase C (PC-PLC). The pore-forming activity of LLO prevents phagosomes from acidification in macrophages, thereby delaying their fusion with lysosomes before mediating phagosome escape [54]. LLO facilitates escape from the phagosome of host cells and protects *L. monocytogenes* from ROS [57]. The upregulation of LLO in conditions of oxidative stress [57] leads to the inhibition of NADPH oxidase, which induces ROS production, ensuring *L. monocytogenes* survival until it escapes from the phagosome [58].

We observed that ROS levels appeared to be unchanged, and a massive number of listeria cells penetrated low dose pre-treated cells. Thus, it is possible to hypothesize that the high levels of LLO, due to the high number of entered bacterial cells, could produce a marked inhibition of cellular ROS production, leading to an increased number of bacterial cells to escape from phagosomes and spread cell-to-cell. Indeed, as interval time increased, we observed that most of the bacteria were free in the cytoplasm and coated with actin, together with a few coated bacteria enclosed in vacuoles. This indicates that the majority of bacteria were able to pass cell-to-cell. On the contrary, at high doses, survival was lower, as visualized by electron microscopy, observing only degrading vacuoles and bacteria-free cytoplasms. In addition to low bacterial uptake, the poor survival of bacteria in cells pre-treated with high TiO_2_ NP dose could also be due to increased oxidative stress induced by NPs, starting at 6 h of treatment. During bacterial entry, *L. monocytogenes* found a high oxidative stress environment, which increased even more after infection, perhaps also due to the low amount of LLO, which was unable to inhibit ROS production into phagosomes.

The effect of TiO_2_ NPs on increasing *Listeria* growth was unexpected, but not without precedent in the literature. Xu et al. (2016) [53] showed that nonactivated TiO_2_ NPs increased the attachment/internalization of *Staphylococcus aureus* into HeLa cells. Moreover, it was demonstrated that exposure of human bronchial epithelial cells to titanium nanosized particles before infection with Respiratory Syncytial Virus (RSV) significantly increased viral replication efficiency [59]. Bogdanov et al. (2017) [60] demonstrated that nonactivated TiO_2_ NPs increased *C. trachomatis* growth in a concentration-dependent manner, with an approximately fourfold increase at 100 μg/mL.

Since the daily intake of TiO_2_ NPs is significant, and their elimination is limited, the interaction of TiO_2_ NPs with the intestinal environment could cause perturbing conditions favorable to pathogenic microorganism infection. Regarding *L. monocytogenes,* it has already been demonstrated that lung tissue modification, induced from exposure to single-walled carbon nanotubes, increased bacterial respiratory infectivity, decreased bacterial phagocytosis by macrophages, and depressed the production of the antimicrobial agent nitric oxide by macrophages [61].

## 5. Conclusions

We evaluated the impact of a low or high dose of TiO_2_ NPs on *L. monocytogenes* infection. Our results demonstrate that the exposure of human intestinal cells to nonactivated TiO_2_ nanosized particles before *L. monocytogenes* infection significantly increases the efficiency of bacterial replication and survival. The most intriguing results were obtained when HT-29 cells were pre-treated with 1 μg/cm^2^ of TiO_2_ NPs followed by exposure to *L. monocytogenes*. Cytoskeletal changes, probably induced by TiO_2_ NP treatment, enhance bacterial internalization. Intracellular bacterial survival also appears higher than in untreated cells, perhaps due to the high levels of listeriolysin O, which protects *L. monocytogenes* from ROS.

Despite all the in vitro cellular model limitations, our results suggest that TiO_2_ NPs exposure to intestinal cells may significantly increase the risk of bacterial infection. Notably, the low dose of NPs, comparable to the real amount of TiO_2_ ingestion through food, results in more risk for human health because a low dose of NPs induces cellular changes favorable to listeria infection.

Elucidate mechanisms by which TiO_2_ NPs enhance susceptibility to infection still need to be investigated. Since gut homeostasis alterations induced by TiO_2_ NPs have already been observed, intestinal conditions promoting a pro-inflammatory environment that could be favorable to bacterial infection should be carefully evaluated as risk assessment of this nanomaterial.

## Figures and Tables

**Table 1:** Size distribution of titanium dioxide nanoparticle (TiO_2_ NP) suspensions in Milli-Q water and Dulbecco’s modified minimum essential medium (DMEM).

Medium	Z-Average (nm)	Peak 1 (nm)	Peak 1 Intensity (%)	Peak 2 (nm)	Peak 2 Intensity (%)	PDI
Milli-Q water	446 ± 11	110 ± 20	2.2 ± 0.8	473 ± 36	16.1 ± 3.5	0.420 ± 0.025
DMEM	1290 ± 79	1120 ± 51	18.6 ±2.1			0.339 ± 0.049

**Table 2:** Surface charge of TiO_2_ NP suspensions in Milli-Q water and DMEM.

Medium	Zeta Potential (mV)
Milli-Q water	−25.8 ± 0.32
DMEM	−8.57 ± 0.98

## References

[1] Samat M.H., Ali A.M.M., Taib F.M., Hassan O.H., Yahya M.Z.A. (2016). Hubbard Ucalculations on optical properties of 3d transition metal oxide TiO_2_. Results Phys..

[2] Abbasi A. (2019). TiO_2_-Based Nanocarriers for Drug Delivery. Nanocarriers for Drug Delivery.

[3] Długosz O., Szostak K., Staroń A., Pulit-Prociak J., Banach M. (2020). Methods for Reducing the Toxicity of Metal and Metal Oxide NPs as Biomedicine. Materials.

[4] Ziental D., Czarczynska-Goslinska B., Mlynarczyk D.T., Glowacka-Sobotta A., Stanisz B., Goslinski T., Sobotta L. (2020). Titanium Dioxide Nanoparticles: Prospects and Applications in Medicine. Nanomaterials.

[5] Bachler G., von Goetz N., Hungerbuhler K. (2015). Using physiologically based pharmacokinetic (PBPK) modeling for dietary risk assessment of titanium dioxide (TiO_2_) nanoparticles. Nanotoxicology.

[6] Chen X.X., Cheng B., Yang Y.X., Cao A., Liu J.H., Du L.J., Liu Y., Zhao Y., Wang H. (2013). Characterization and preliminary toxicity assay of nano-titanium dioxide additive in sugar-coated chewing gum. Small.

[7] Dudefoi W., Terrisse H., Popa A.F., Gautron E., Humbert B., Ropers M.H. (2018). Evaluation of the content of TiO_2_ nanoparticles in the coatings of chewing gums. Food Addit. Contam. Part A Chem. Anal. Control EXPO Risk Assess.

[8] EFSA ANS Panel (2016). Scientific opinion on the re-evaluation of titanium dioxide (E 171) as a food additive. EFSA J..

[9] Peters R.J., van Bemmel G., Herrera-Rivera Z., Helsper H.P., Marvin H.J., Weigel S., Tromp P.C., Oomen A.G., Rietveld A.G., Bouwmeesterm H. (2014). Characterization of titanium dioxide nanoparticles in food products: Analytical methods to define nanoparticles. J. Agric. Food Chem..

[10] Baan R., Straif K., Grosse Y., Secretan B., El Ghissassi F., Cogliano V. (2006). Carcinogenicity of carbon black, titanium dioxide, and talc. Lancet Oncol..

[11] IARC Monographs on the Evaluation of Carcinogenic Risks to Humans (2010). Carbon Black, Titanium Dioxide, and Talc. Lyon, France. https://monographs.iarc.fr/wp-content/uploads/2018/06/mono93.pdf.

[12] Wang J., Liu Y., Jiao F., Lao F., Li W., Gu Y., Li Y., Ge C., Zhou G., Li B. (2008). Time-dependent translocation and potential impairment on central nervous system by intranasally instilled TiO_2_ nanoparticles. Toxicology.

[13] McClements D.J., DeLoid G., Pyrgiotakis G., Shatkin J.A., Xiao H., Demokritou P. (2016). The role of the food matrix and gastrointestinal tract in the assessment of biological properties of ingested engineered nanomaterials (iENMs): State of the science and knowledge gaps. NanoImpact.

[14] Baranowska-Wójcik E., Szwajgier D., Oleszczuk P., Winiarska-Mieczan A. (2019). Effects of titanium dioxide nanoparticles exposure on human health-a review. Biol. Trace Elem. Res..

[15] Brun E., Barreau F., Veronesi G., Fayard B., Sorieul S., Chanéac C., Carapito C., Rabilloud T., Mabondzo A., Herlin-Boime N. (2014). Titanium dioxide nanoparticle impact and translocation through ex vivo, in vivo and in vitro gut epithelia. Part. Fibre Toxicol..

[16] Nogueira C.M., de Azevedo W.M., Dagli M.L., Toma S.H., Leite A.Z., Lordello M.L., Nishitokukado I., Ortiz-Agostinho C.L., Duarte M.I., Ferreira M.A. (2012). Titanium dioxide induced inflammation in the small intestine. World J. Gastroenterol..

[17] Ammendolia M.G., Iosi F., Maranghi F., Tassinari R., Cubadda F., Aureli F., Raggi A., Superti F., Mantovani A., De Berardis B. (2017). Short-term oral exposure to low doses of nano-sized TiO_2_ and potential modulatory effects on intestinal cells. Food Chem. Toxicol..

[18] Wang J., Zhou G., Chen C., Yu H., Wang T., Ma Y., Jia G., Gao Y., Li B., Sun J. (2007). Acute toxicity and biodistribution of different sized titanium dioxide particles in mice after oral administration. Toxicol. Lett..

[19] Hong F., Yu X., Wu N., Zhang Y.Q. (2017). Progress of in vivo studies on the systemic toxicities induced by titanium dioxide nanoparticles. Toxicol. Res..

[20] Ze Y., Sheng L., Zhao X., Hong J., Ze X., Yu X., Pan X., Lin A., Zhao Y., Zhang C. (2014). TiO_2_ nanoparticles induced hippocampal neuroinflammation in mice. PLoS ONE.

[21] Tassinari R., Cubadda F., Moracci G., Aureli F., D’Amato M., Valeri M., De Berardis B., Raggi A., Mantovani A., Passeri D. (2014). Oral, short-term exposure to titanium dioxide nanoparticles in Sprague-Dawley rat: Focus on reproductive and endocrine systems and spleen. Nanotoxicology.

[22] Györgyey Á., Janovák L., Ádám A., Kopniczky J., Tóth K.L., Deák Á., Panayotov I., Cuisinier F., Dékány I., Turzó K. (2016). Investigation of the in vitro photocatalytic antibacterial activity of nanocrystalline TiO_2_ and coupled TiO_2_/Ag containing copolymer on the surface of medical grade titanium. J. Biomater. Appl..

[23] Adams L.K., Lyon D.Y., Alvarez P.J.J. (2006). Comparative eco-toxicity of nanoscale TiO_2_, SiO_2_, and ZnO water suspensions. Water Res..

[24] Heinlaan M., Ivask A., Blinova I., Dubourguier H.C., Kahru A. (2008). Toxicity of nanosized and bulk ZnO, CuO and TiO_2_ to bacteria vibrio fischeri and crustaceans daphnia magna and Thamnocephalus platyurus. Chemosphere.

[25] Zhang X.C., Li W., Yang Z. (2015). Toxicology of nanosized titanium dioxide:an update. Arch. Toxicol..

[26] Alizadeh-Sani M., Hamishehkar H., Khezerlou A., Maleki M., Azizi-Lalabadi M., Bagheri V., Safaei P., Azimi T., Hashemi M., Ehsani A. (2020). Kinetics Analysis and Susceptibility Coefficient of the Pathogenic Bacteria by Titanium Dioxide and Zinc Oxide Nanoparticles. Adv. Pharm. Bull..

[27] Farber J.M., Peterkin P.I. (1991). Listeria monocytogenes, a food-borne pathogen. Microbiol. Rev..

[28] Ramaswamy V., Cresence V.M., Rejitha J.S., Lekshmi M.U., Dharsana K.S., Prasad S.P., Vijila H.M. (2007). Listeria--review of epidemiology and pathogenesis. J. Microbiol. Immunol. Infect..

[29] Swaminathan B., Gerner-Smidt P. (2007). The epidemiology of human listeriosis. Microbes Infect..

[30] Rolhion N., Cossart P. (2017). How the study of Listeria monocytogenes has led to new concepts in biology. Future Microbiol..

[31] Loepfe C., Raimann E., Stephan R., Tasara T. (2010). Reduced host cell invasiveness and oxidative stress tolerance in double and triple csp gene family deletion mutants of Listeria monocytogenes. Foodborne Pathog. Dis..

[32] Ammendolia M.G., Iosi F., De Berardis B., Guccione G., Superti F., Conte M.P., Longhi C. (2014). Listeria monocytogenes behaviour in presence of non-UV-irradiated titanium dioxide nanoparticles. PLoS ONE.

[33] Glaser P., Frangeul L., Buchrieser C., Rusniok C., Amend A., Baquero F., Berche P., Bloecker H., Brandt P., Chakraborty T. (2001). Comparative genomics of Listeria species. Science.

[34] Alonzo F., Port G.C., Cao M., Freitag N.E. (2009). The post-translocation chaperone PrsA2 contributes to multiple facets of Listeria monocytogenes pathogenesis. Infect. Immun..

[35] Verleysen E., Waegeneers N., Brassinne F., De Vos S., Jimenez I.O., Mathioudaki S., Mast J. (2020). Physicochemical characterization of the Pristine E171 food Additive by standardized and validated methods. Nanomaterials.

[36] Calzolai L., Gilliland D., Rossi F. (2012). Measuring nanoparticles size distribution in food and consumer products: A review. Food Addit. Contam. Part A Chem. Anal. Control EXPO Risk Assess.

[37] Rompelberg C., Heringa M.B., van Donkersgoed G., Drijvers J., Roos A., Westenbrink S., Peters R., van Bemmel G., Brand W., Oomen A.G. (2016). Oral intake of added titanium dioxide and its nanofraction from food products, food supplements and toothpaste by the Dutch population. Nanotoxicology.

[38] Sprong C., Bakker M., Niekerk M., Vennemann F. (2016). Exposure Assessment of the Food Additive Titanium Dioxide (E 171) Based on Use Levels Provided by the Industry.

[39] Weir A., Westerhoff P., Fabricius L., Hristovski K., von Goetz N. (2012). Titanium dioxide nanoparticles in food and personal care products. Environ. Sci. Technol..

[40] Montiel-Davalos A., Ventura-Gallegos J.L., Alfaro-Moreno E., Soria-Castro E., Garcia-Latorre E., Cabañas-Moreno J.G., del Pilar Ramos-Godinez M., López-Marure R. (2012). TiO_2_ nanoparticles induce dysfunction and activation of human endothelial cells. Chem. Res. Toxicol..

[41] Rueda-Romero C., Hernández-Pérez G., Ramos-Godínez P., Vázquez-López I., Quintana-Belmares R.O., Huerta-García E., Stepien E., López-Marure R., Montiel-Dávalos A., Alfaro-Moreno E. (2016). Titanium dioxide nanoparticles induce the expression of early and late receptors for adhesion molecules on monocytes. Part. Fibre Toxicol..

[42] Mengaud J., Ohayon H., Gounon P., Mege R.M., Cossart P. (1996). E-cadherin is the receptor for internalin, a surface protein required for entry of L. monocytogenes into epithelial cells. Cell.

[43] Wollert T., Paschem B., Rochon M., Deppenmeier S., van den Heuvel J., Gruber A.D., Heinz D.W., Lengeling A., Schubert W.D. (2007). Extending the host range of Listeria monocytogenes by rational protein design. Cell.

[44] Ortega F.E., Rengarajan M., Chavez N., Radhakrishnan P., Gloerich M., Bianchini J., Siemers K., Luckett W.S., Lauer P., Nelson W.J. (2017). Adhesion to the host cell surface is sufficient to mediate Listeria monocytogenes entry into epithelial cells. Mol. Biol. Cell..

[45] Koeneman B.A., Zhang Y., Westerhoff P., Chen Y., Crittenden J.C., Capco D.G. (2010). Toxicity and cellular responses of intestinal cells exposed to titanium dioxide. Cell Biol. Toxicol..

[46] Simon M., Barberet P., Delville M.H., Moretto P., Seznec H. (2011). Titanium dioxide nanoparticles induced intracellular calcium homeostasis modification in primary human keratinocytes. Towards an in vitro explanation of titanium dioxide nanoparticles toxicity. Nanotoxicology.

[47] Martin-Romero F.J., Lopez-Guerrero A.M., Pascual-Caro C., Pozo-Guisado E., Jimenez-Lopez J.C. (2017). The Interplay between Cytoskeleton and Calcium Dynamics, Cytoskeleton. Structure, Dynamics, Function and Disease.

[48] Simon M., Saez G., Muggiolu G., Lavenas M., Le Trequesser Q., Michelet C., Devès G., Barberet P., Chevet E., Dupuy D. (2017). In situ quantification of diverse titanium dioxide nanoparticles unveils selective endoplasmic reticulum stress-dependent toxicity. Nanotoxicology.

[49] Banerjee S., Gopal J., Muraleedharan P., Tyagi A.K., Rai B. (2006). Physics and chemistry of photocatalytic titanium dioxide: Visualization of bactericidal activity using atomic force microscopy. Curr. Sci..

[50] Thevenot P., Cho J., Wavhal D., Timmons R.B., Tang L.P. (2008). Surface chemistry influences cancer killing effect of TiO_2_ nanoparticles. Nanomedicine.

[51] Da Rosa E.L. (2013). Kinetic effects of TiO_2_ fine particles and nanoparticles aggregates on the nanomechanical properties of human neutrophils assessed by force spectroscopy. BMC Biophys..

[52] Tran T.H., Nosaka A.Y., Nosaka Y. (2006). Adsorption and photocatalytic decomposition of amino acids in TiO_2_ photocatalytic systems. J. Phys. Chem. B.

[53] Xu Y., Wei M.T., Ou-Yang H.D., Walker S.G., Wang H.Z., Gordon C.R., Guterman S., Zawacki E., Applebaum E., Brink P.R. (2016). Exposure to TiO_2_ nanoparticles increases Staphylococcus aureus infection of HeLa cells. J. Nanobiotechnol..

[54] Shaughnessy L.M., Hoppe A.D., Christensen K.A., Swanson J.A. (2006). Membrane perforations inhibit lysosome fusion by altering pH and calcium in Listeria monocytogenes vacuoles. Cell. Microbiol..

[55] Birmingham C.L., Higgins D.E., Brumell J.H. (2008). Avoiding death by autophagy: Interactions of Listeria monocytogenes with the macrophage autophagy system. Autophagy.

[56] Py B.F., Lipinski M.M., Yuan J. (2007). Autophagy limits Listeria monocytogenes intracellular growth in the early phase of primary infection. Autophagy.

[57] Lam G.Y., Fattouh R., Muise A.M., Grinstein S., Higgins D.E., Brumell J.H. (2011). Listeriolysin O suppresses phospholipase C-mediated activation of the microbicidal NADPH oxidase to promote Listeria monocytogenes infection. Cell Host Microbe.

[58] David R. (2012). Bacterial pathogenesis: A balancing act for LLO and PLC. Nat. Rev. Microbiol..

[59] Chakraborty S., Castranova V., Perez M.K., Piedimonte G. (2017). Nanoparticles increase human bronchial epithelial cell susceptibility to respiratory syncytial virus infection via nerve growth factor-induced autophagy. Physiol. Rep..

[60] Bogdanov A., Janovák L., Lantos I., Endrész V., Sebők D., Szabó T., Dékány I., Deák J., Rázga Z., Burián K. (2017). Nonactivated titanium-dioxide nanoparticles promote the growth of Chlamydia trachomatis and decrease the antimicrobial activity of silver nanoparticles. J. Appl. Microbiol..

[61] Shvedova A.A., Fabisiak J.P., Kisin E.R., Murray A.R., Roberts J.R., Tyurina Y.Y., Antonini J.M., Feng W.H., Kommineni C., Reynolds J. (2008). Sequential exposure to carbon nanotubes and bacteria enhances pulmonary inflammation and infectivity. Am. J. Respir. Cell Mol. Biol..

